# Nucleic Acid Testing for the Detection of HBV DNA

**DOI:** 10.5812/kowsar.1735143X.780

**Published:** 2011-10-01

**Authors:** Wai Kay Seto, Ching Lung Lai, Man Fung Yuen

**Affiliations:** 1 Department of Medicine,University of Hong Kong, Queen Mary Hospital, Hong Kong, China

**Keywords:** Nucleic Acids, Hepatitis B Virus, DNA

Dear Editor,

We read with interest the article by Arababadi et al. [1], which highlights different and important areas of concern in the management of occult hepatitis B (OBI). There are two relevant issues worthy of discussion. First, the authors described the variable prevalence of OBI in different geographical regions worldwide. The laboratory assay used to detect OBI is an important determinant of its prevalence. Because of the low viremic state of patients, OBI may not be detected even when sensitive polymerase chain reaction (PCR) assays are employed. Results of a recent population study in our locality involving 13,034 blood donors showed the prevalence of OBI to be between 0.11% and 0.13% [2]. We used nucleic acid testing to detect hepatitis B virus (HBV) DNA, which has a detection limit (3.7 IU/mL) lower than that of most available PCR assays. Only studies using sensitive assays should be considered when implementing policies for screening blood donors. Although Arababadi et al. have suggested using an antibody against the hepatitis B core antigen (anti-HBc) for screening blood donors, there is a vast difference in the prevalence of serum anti-HBc positivity and OBI worldwide. For example, the preva-lence of anti-HBc positivity in Korea is 13.5%, whereas the prevalence of OBI in the same region is 0.016% [3]. Hence, in blood donor screening programs, hepatitis B surface antigen (HBsAg) testing followed by nucleic acid testing should be regarded as the most accurate approach for the detection of OBI.

Second, the authors have suggested that OBI is a possible risk factor for hepatocellular carcinoma (HCC), but they have also commented that the underlying patho-genesis is unclear. Recently, an Asian study compared the tumorous and adjacent non-tumorous liver tissues from 33 cases of cryptogenic HCC and 28 of HCC with identifiable causes [4]. Both intrahepatic HBV DNA and HBV pregenomic RNA (pgRNA) were detected in 73% of the cases of cryptogenic HCC, albeit at levels lower than those detected in cases of HBV-related HCC. Hence, it is very likely that both persistent viral replication and viral integration play an important role in the pathogenesis of OBI-related HCC. The detection rate of intrahepatic HBV DNA was higher in the non-tumorous tissues than in the tumorous tissues; this finding is consistent with the hypothesis that tumorous tissues lack the optimal environment for HBV replication [5], which may affect the detection of OBI-related HCC. Therefore, in regions with a high prevalence of chronic hepatitis B, OBI should be considered as a possible cause of cryptogenic HCC even when HBV virologic markers are undetectable in tumor histological analysis.
